# Fibula response to disuse: a longitudinal analysis in people with spinal cord injury

**DOI:** 10.1007/s11657-022-01095-9

**Published:** 2022-03-19

**Authors:** Shima Abdelrahman, Mariel Purcell, Timo Rantalainen, Sylvie Coupaud, Alex Ireland

**Affiliations:** 1 Department of Biomedical Engineering, Wolfson Building, University of Strathclyde, Glasgow, UK; 2 Scottish Centre for Innovation in Spinal Cord Injury, Queen Elizabeth National Spinal Injuries Unit, Queen Elizabeth University Hospital, Glasgow, UK; 3 Research Centre for Musculoskeletal Science & Sports Medicine, Department of Life Sciences, Manchester Metropolitan University, Manchester, UK; 4 Neuromuscular Research Center, Department of Biology of Physical Activity, University of Jyvaskyla, Jyvaskyla, Finland

**Keywords:** Fibula, Disuse osteoporosis, Mechanoadaptation, Spinal cord injury, pQCT

## Abstract

***Summary*:**

Fibular response to disuse has been described in cross-sectional but not longitudinal studies. This study assessed fibular bone changes in people with spinal cord injury. Fibular bone loss was less than in the tibia and was not correlated together. This might explain low fibular fracture incidents in these patients.

**Purpose:**

Cross-sectional studies suggest that the fibula responds differently to loading and disuse compared to the tibia. Whilst tibial bone changes following spinal cord injury (SCI) have been established in longitudinal studies, fibular changes remain unexplored.

**Methods:**

Fibular and tibial bone parameters were assessed in 13 individuals with SCI (aged 16–76 years). Peripheral quantitative computed tomography scans were acquired at 4%, 38% and 66% distal–proximal tibia length at 5 weeks and 12 months post-injury. Changes in 4% site total bone mineral content (BMC), total cross-sectional area (CSA) and bone mineral density (BMD), and 38% and 66% sites total BMC, total CSA, cortical BMD and cortical CSA were assessed using paired *T*-tests. Relationships between bone loss in the two bones at equivalent sites were assessed using paired *T*-tests and correlation.

**Results:**

At the 4% site, fibular total BMC and BMD losses were less than tibial losses (− 6.9 ± 5.1% and − 6.6 ± 6.0% vs − 14.8 ± 12.4% and − 14.4 ± 12.4%, *p* = 0.02 and *p* = 0.03, respectively). Similarly, at the 66% site, fibular BMC losses were less than those in the tibia (− 2.0 ± 2.6% vs − 4.3 ± 3.6%, *p* = 0.03), but there was no difference at 38% (− 1.8 ± 3.5% vs − 3.8 ± 2.1%, *p* = 0.1). No correlation was observed for BMC changes between the two bones (all *p* > 0.25).

**Conclusion:**

These results support cross-sectional evidence of smaller disuse-related bone loss in the fibula compared to the tibia. These results may in part explain lower incidence of fibula fractures in individuals with chronic SCI. The lack of association between losses in the two bones might point to different underlying mechanisms.

## Introduction

The human fibula has a much smaller cross-sectional area and lower bone mineral content compared to the neighbouring tibia [1]. It supports only 5–19% of the shank axial loading, but this load proportion increases with load magnitude[2].

The tibia increases in size and density after harvesting of the fibula for humerus reconstruction [3], emphasising the important mechanical role that fibula plays. Moreover, the greater fibular strength (for lateral bending) in soccer players compared to untrained controls and the dramatic increase in the size of the fibula after transplanting it to replace an excised tibia [4] further indicate that the fibula has the capacity to adapt to an altered loading environment. However, this adaptation seems to be different in training [5] and disuse [6] compared to the tibia.

Cross-sectional studies suggest that fibula bone loss following spinal cord injury (SCI) is modest and confined to epiphyseal regions [6], whereas the tibia undergoes extensive loss along its length [7, 8]. However, to date, the fibula’s response to disuse has not been explored in longitudinal studies. The absence of disuse-related loss could help explain the low incidence of fibula fractures in individuals with SCI (approximately 1/5 of the number of fractures reported in the femur and tibia) [9]. In addition, greater understanding of the fibula shaft’s apparent protection from disuse-related losses could lead to strategies to prevent loss in other bone regions.

Therefore, in this study, fibular and tibial bone parameters were assessed and compared in individuals with SCI within 12 months following their injury. Our aim was to describe longitudinal disuse-related changes in the fibula during the first 12 months of SCI and to compare these changes to those in the tibia. We hypothesised that, in line with previous cross-sectional observations, bone loss in the fibula would be much smaller than that in the tibia and only evident in the epiphysis.

## Methods

Twenty-nine inpatients (aged 16–76 years) with motor-complete SCI (grades A or B on the American Spinal Injuries Association Impairment Scale (AIS)) at the Queen Elizabeth National Spinal Injuries Unit (UK) were recruited for this study. Longitudinal changes in the tibia in these individuals have been reported previously [10]. The main exclusion criteria were age < 16 years; recent bone fracture and continued ventilator dependency at week 5 post-injury. Ethical approval for the study was obtained from the NHS Research Ethics Committee. Further details on patient recruitment and scanning protocols for that study have been described previously [10].

pQCT scans (XCT3000, Stratec Medizintechnik GmbH, Germany) were obtained by a single operator from these 29 participants within the first 5 weeks (baseline) and at 4, 8 and 12 months post-injury and were analysed for longitudinal changes in bone parameters at the tibia throughout the first year of injury [10]. Of these, a subgroup of 13 individuals with complete sets of baseline and 12-month scans was included in this study, as these were the only two timepoints considered in this investigation of the fibula.

Scans of the tibia and fibula were taken at 4%, 38% and 66% of tibial length (from the distal reference point). These scans were analysed using an ImageJ plugin (National Institutes of Health, Maryland, USA) [11, 12]. Epiphyseal parameters calculated at the 4% site were the total bone mineral content (BMC), total cross-sectional area (CSA) and bone mineral density (BMD). Total rather than trabecular BMD was examined at this site due to the thick cortex and small trabecular area evident at distal fibula sites [6]. The parameters calculated at 38% and 66% (diaphyseal) sites were total BMC, total CSA, cortical BMD and cortical CSA. Given the thin cortex in individuals with SCI at epiphyseal sites, thresholds of 120 mg·mm^−3^ and 150 mg·mm^−3^ were used to separate bone and soft tissue at the epiphyseal and diaphyseal sites, respectively. Short-term error of tibia and fibula pQCT scans assessed using similar thresholds in paired scans from twenty-five individuals by our group was very low. In both bones, coefficient of variation of total BMC was less than 0.6% whereas for no other parameter was this value greater than 1.5%.

The normality of the data has been assessed using the Anderson–Darling normality test. Where data were normally distributed, parametric tests were performed to assess changes in all bone parameters Where data were not normally distributed, non-parametric Wilcoxon signed rank test was used. Analysis was performed on Minitab Statistical software (Minitab, version 19). In addition both absolute and relative changes in the same bone variables in tibia and fibula were compared using paired *T*-tests, with relative changes calculated as the percentage change from baseline. In addition, relationships between normalised (%) bone loss in the two bones at the equivalent site were assessed using Spearman correlation, paired *T*-tests and Wilcoxon signed test (for tibial BMD at 4%).

## Results

Descriptive statistics of bone parameters at baseline and 12 months post-injury at 4%, 38% and 66% of tibial length in fibula and tibia are summarised in Table 1. All the data were normally distributed, with the exception of the tibial total BMD at the 4% site.

**Table 1:** Descriptive statistics (mean, standard deviation (SD), median, interquartile range (IQR)) of bone parameters at baseline and 12 months post-injury at 4%, 38% and 66% of tibial length in fibula and tibia

Bone	Fibula									Tibia							
Scan site and parameter																	
	Baseline				12 months					Baseline				12 months			
	Mean	SD	Median	IQR	Mean	SD	Median		IQR	Mean	SD	Median	IQR	Mean	SD	Median	IQR
Distal tibia 4%																	
BMC (mg/mm)	104.5	26.9	100.8	45.7	97.3	25.6	95.9		41.8	418.1	50.1	420.9	50.3	356.9	72.1	378.5	106.6
BMD (mg/cm^3^)	562.2	92.6	580.2	110.8	525.4	94.1	524.7		124.5	-	-	334.5	30.4	-	-	301.0	60.9
Total CSA (mm^2^)	186.2	36.9	184.5	57.4	186.7	41.6	177.8	56.1		1268.4	137.6	1290.8	167.1	1263.6	149.1	1257.5	170.4
Distal tibia 38%																	
BMC (mg/mm)	120.6	19.9	119.3	37.1	118.4	20.2	117.8	41.5		431.3	55.2	439.7	63.2	415.2	57.7	415.4	58.6
Cortical BMD (mg/cm^3^)	892.8	36.7	899.2	50.3	892.6	38.6	879.4	68.0		971.8	28.7	983.7	41.2	947.3	27.3	957.6	53.0
Total CSA (mm^2^)	154.0	28.5	155.5	53.5	151.6	28.1	151.5	51.8		509.1	57.7	500.5	72.3	504.2	57.6	499.8	73.3
Cortical CSA (mm^2^)	134.3	22.5	130.3	42.5	131.8	21.9	129.5	40.4		440.0	53.0	442.5	47	434.1	53.7	431	43.5
Distal tibia 66%																	
BMC (mg/mm)	101.2	17.6	101.7	34.1	99.2	17.7	97.6	34.0		480.0	57.5	474.2	75.8	459.3	59.4	449.1	84.8
Cortical BMD (mg/cm^3^)	834.6	52.5	835.0	80.4	842.8	59.5	853.7	67.3		834.9	49.1	839.6	66.0	811.8	57.9	801.4	62.6
Total CSA (mm^2^)	129.4	22.6	134.5	36.8	126.8	23.3	130.3	38.1		763.8	100.1	765.0	158.8	760.7	96.9	775.9	168.6
Cortical CSA (mm^2^)	120.4	19.5	123.5	31.6	117.1	20.3	119.1	36.4		564.3	66.2	560.3	100.8	553.4	60.7	538.6	89.6

**Fig. 1:**
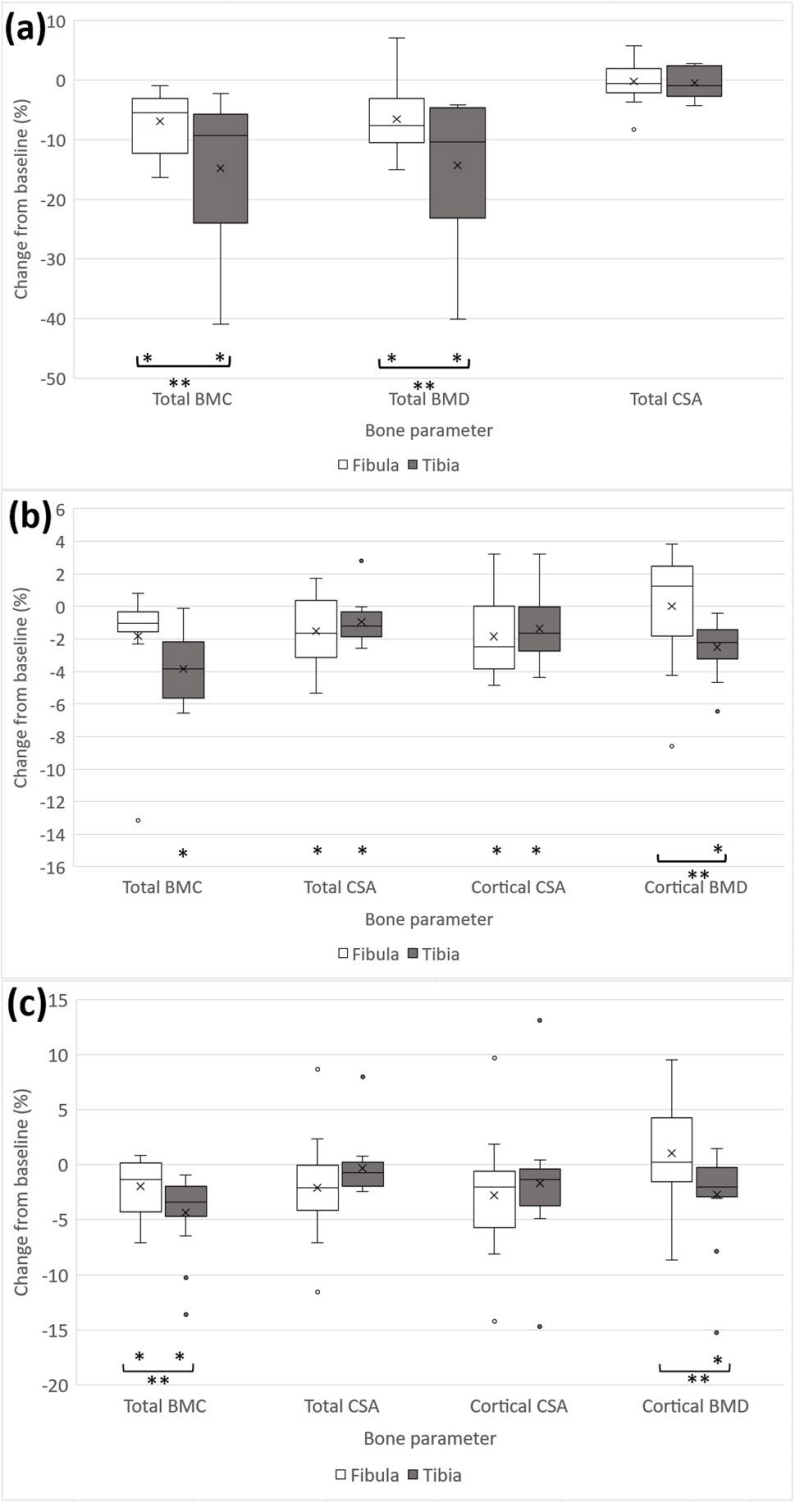
Box plots of change at 12 months post-injury relative to baseline values in: **a** Total bone mineral content (BMC), total bone mineral density (BMD), and total area (CSA) at the distal 4%, **b** total BMC, total CSA, cortical CSA and cortical BMD at 38% site, **c** total BMC, total CSA, cortical CSA and cortical BMD at 66% site (distal to proximal) of tibia and fibula bones. (*) Indicates significant change in bone parameter at 12 months, (**) Indicates significant difference between tibial and fibular percentage changes

At the 4% site, both total BMC and BMD declined in both bones over the time-course of observation. However, tibial BMC and BMD losses (− 14.8 ± 12.4% and − 14.4 ± 12.4%, both *p* < 0.05) were greater than those observed in the fibula (− 6.9 ± 5.1% and − 6.6 ± 6.0%, *p* = 0.02 and *p* = 0.03, respectively). Both bones maintained their total area at this site (*p* = 0.6 for the tibia; *p* = 0.8 for the fibula) (Fig. 1(a)).

**Fig. 2:**
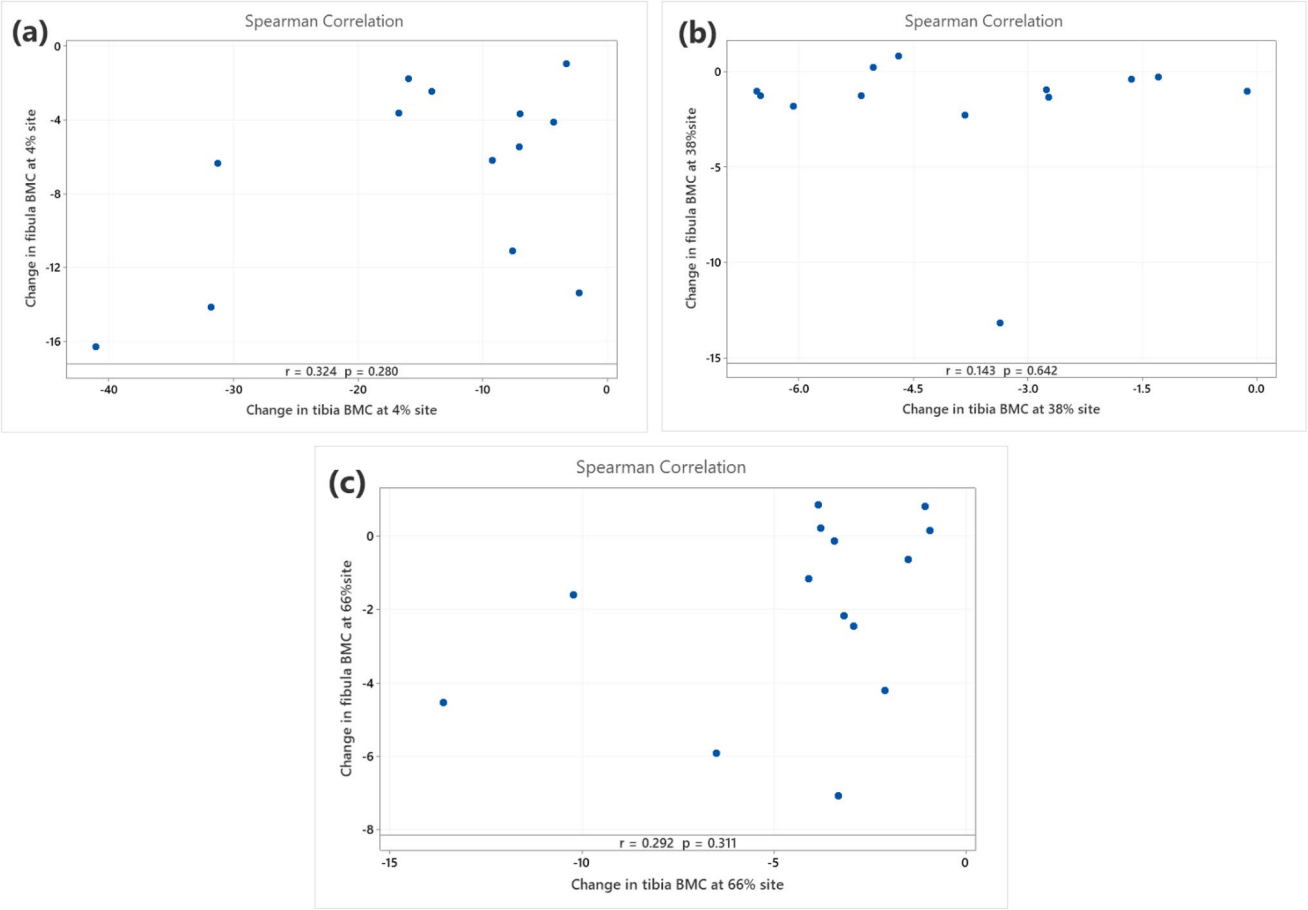
Matrix plots of correlation results of changes in total bone mineral content (BMC) between tibia and fibula at **a** 4%, **b** 38% and **c** 66% of tibial length between baseline and 12 months post-injury

In the diaphyses, BMC decreased at both tibial sites (38%, 66%), with tibial losses at 66% being twice those in the fibula (− 4.3 ± 3.6% vs − 2.0 ± 2.6%, *p* = 0.03). However, whilst fibular losses were similar in magnitude at both sites, the statistical evidence of a loss at 38% was weak (*p* = 0.06), and there was no evidence of a difference in loss between the tibia and fibula (− 3.8 ± 2.1% vs − 1.8 ± 3.5%, *p* = 0.1), partly due to the larger standard deviation (as can be seen in Fig. 1(b, c)). Differences in BMC loss resulted from greater cortical BMD losses in tibia than fibula at both sites (− 2.5 ± 1.6% vs 0.0 ± 3.6% and − 2.7 ± 4.2% vs 1.03 ± 4.7%, *p* = 0.01 and *p* = 0.02, respectively). In contrast, whilst CSA and cortical CSA decreased in both tibia and fibula at the 38% site (− 0.9 ± 1.4% vs − 1.5 ± 2.1% and − 1.4 ± 1.9% vs − 1.8 ± 2.4%, *p* = 0.5 and *p* = 0.63, respectively) and were maintained at the 66% site in both bones (− 0.3 ± 2.6% vs − 2.1 ± 4.7% and − 1.7 ± 5.7% vs − 2.8 ± 5.4%, *p* = 0.07 and *p* = 0.4, respectively), these changes were similar in both bones. No evidence of a correlation was found between changes in BMC between the two bones (all *p* > 0.25; Fig. 2).

In secondary analyses, we investigated whether the relative proportions of cortical and trabecular bones could contribute to observed differences in bone loss between the tibia and fibula. In the distal tibia, cortical and trabecular bones made up 69 ± 3% and 31 ± 3% of total BMC, respectively (full data not shown). Whilst percentage bone loss from the cortical component (18 ± 6%) was slightly larger than from the trabecular component (14 ± 7%), this difference was not significant (*p* = 0.32). In contrast, bone at the distal fibula was mostly cortical (99.7 ± 0.7%), and losses from this component (7.2 ± 5.6%) were smaller than those observed at the distal tibia (*p* < 0.001). As expected, at both tibia and fibula diaphyseal sites the bone was almost entirely cortical, with > 98% cortical content at all sites.

## Discussion

The aim of this study was to describe longitudinal changes in the fibula bone in response to disuse during the first 12 months of SCI and to compare these changes to those in the tibia. Across epiphyseal and diaphyseal sites, fibular bone loss was less than 50% of that at the corresponding tibia site which supports the results from previous cross-sectional studies [6, 13, 14]. Losses in the tibia and fibula within each participant were not correlated with each other. The loss of BMC that was evident at the fibular shaft (38% and 66%, respectively) contrasts with a previous cross-sectional report that observed no difference in BMC in the fibular shaft in chronic SCI [6]. In both the fibula and tibia, bone losses were more prominent at the distal end compared to the shaft, which supports findings from cross-sectional studies [15, 16].

Previous evidence suggests that: (i) relative changes in fibula loading are greater than those in the tibia, (ii) the fibula supports a substantial portion of shank loading during physical activity and (iii) the fibula is able to change its size and mass dramatically in response to increased loading [2–4]. Therefore, it is perhaps surprising that disuse-related bone losses are less than half those in the neighbouring bone. In addition, the lack of correlation reported here between the tibia and fibula suggests further that they are affected by different mechanisms. Evidence for (ii) and (iii) could be considered robust, particularly for (ii) when we consider the occurrence of fibula stress fractures in athletes. However, proposition (i) is based on cadaveric data and, to date, the in vivo loading environment of the fibula is unknown. In addition, previous data describe static loading conditions, and it is well established that the rate of force application is a key determinant of bone mechanoadaptive response. Therefore, assessment of fibula deformation in vivo would improve our understanding of fibula’s mechanoadaptive response.

The mechanisms leading to less pronounced bone loss in the fibula compared to the tibia are not fully understood, but structural differences between the two bones have been considered. The endocortical surface, with its higher rate of bone turnover is larger in the tibia, and previous studies showed that between-site differences in endocortical circumference are strongly correlated with site-specific loss in the tibia [15, 8]. However, when normalised to bone size the surface:area ratio is greater in the fibula than in the equivalent sites in the tibia suggesting that this does not contribute to observed inter-bone differences. For the two diaphyseal sites, the percentage loss was identical. However, it was statistically evident at the 66% and not at the 38% due to the greater dispersion in BMC changes at 38% which appeared to be related to one outlier.

Divergent responses of the distal tibia and fibula to disuse could alternatively be explained in part by the greater trabecular component in the distal tibia, that is known to show a more rapid response to disuse (in absolute terms) compared to the cortical component [7, 16]. In secondary analyses, we considered the relative proportions of trabecular and cortical bone in addition to relative losses in the two bone regions. At the distal tibia, 31% of bone mass was trabecular whereas the proportion was negligible in the fibula. However, percentage bone loss was higher in the distal tibia cortical component than in the distal fibula, and these losses were also more than twice as large as those observed in the cortical component of the distal fibula. When considering that at both tibia and fibula shaft sites the bone was almost entirely cortical, it seems clear that the relative proportions of trabecular and cortical bone cannot explain the differences in bone loss between the tibia and fibula at any site. Whilst caution must be used when assessing cortical bone at the distal tibia using pQCT due to the thin cortical shell and associated partial volume effect, it is reassuring that our findings of similar loss in cortical and trabecular components is similar to a previous report using high-resolution pQCT [14].

That modest fibula response to disuse may explain the low incidence of fibula fractures in patients with SCI, who tend to experience fragility fractures mostly in the distal femur and tibial epiphyses [17]. Moreover, a deeper understanding of the mechanisms that lead to these smaller fibular deficits in disuse could help us develop therapies to mitigate or treat osteoporosis. Understanding these different responses to disuse can also provide more insights into neuro-skeletal interactions that are yet to be fully understood.

To the best of our knowledge, this study is the first to explore the fibula’s response to disuse following SCI longitudinally. Within-individual comparisons enabled a characterisation of disuse-related loss in the fibula which has not been possible in previous cross-sectional studies. However, the absence of an uninjured control group in this longitudinal study, prevented a direct comparison to determine whether the bone losses observed in the fibula differed from typical age-related changes. Whilst tibial changes are clearly far greater than those observed in controls [18], no comparable data exists for the fibula. In the only longitudinal study of the fibula bone in older adult athletes (in whom disuse does not contribute), the fibula changes in the shaft are not entirely dissimilar [19]. Therefore, further controlled studies or alternative disuse models should be examined.

## Conclusions

Fibula bone losses following SCI are less pronounced than in the neighbouring tibia. This is despite the substantial contribution which the fibula makes to shank loading, and evidence that the fibula has the capacity to adapt in response to increased loading. The losses in the two bones are seemingly not related, suggesting that they may be influenced by different mechanisms. Alternatively, the differences in their mechanical loading in vivo which have not been revealed by previous ex vivo studies may contribute. In contrast to previous cross-sectional reports, some loss of bone mass was observed in the fibula. These results may in part explain lower incidence of fibula fractures in individuals with chronic SCI. Further study of the biomechanics of the two bones is required.

## Data Availability

The bone scans and datasets produced and analyzed during this study are available from the authors.
